# Chemical Composition, Fatty Acid Profile and Sensory Characteristics of Chanco-Style Cheese from Early Lactation Dairy Cows Fed Winter Brassica Crops

**DOI:** 10.3390/ani11010107

**Published:** 2021-01-07

**Authors:** Einar Vargas-Bello-Pérez, Carolina Geldsetzer-Mendoza, Rodrigo A. Ibáñez, José Ramón Rodríguez, Christian Alvarado-Gillis, Juan P. Keim

**Affiliations:** 1 Department of Veterinary and Animal Sciences, Faculty of Health and Medical Sciences, University of Copenhagen, Grønnegårdsvej 3, DK-1870 Frederiksberg C, Denmark; 2 Departamento de Ciencias Animales, Facultad de Agronomía e Ingeniería Forestal, Pontificia Universidad Católica de Chile, Macul, Santiago 7820436, Chile; c.geldsetzer@gmail.com (C.G.-M.); ribanez@cdr.wisc.edu (R.A.I.); 3 Center for Dairy Research, University of Wisconsin-Madison, Madison, WI 53706, USA; 4 Escuela de Graduados, Facultad de Ciencias Agrarias y Alimentarias, Universidad Austral de Chile, Independencia 631, Valdivia 5110566, Chile; jrrodriguezr119@yahoo.es; 5 Facultad de Ciencias Agrarias y Alimentarias, Instituto de Producción Animal, Universidad Austral de Chile, Independencia 631, Valdivia 5110566, Chile; calvarado@uach.cl

**Keywords:** biohydrogenation, milk, fatty acids, cheese, brassicas

## Abstract

**Simple Summary:**

Brassica crops such as kales and swedes can be supplied to cow diets during winter. Little is known about the effects of feeding those forage brassicas to lactating cows on cheese nutritional characteristics. Thus, the objective of this study was to determine the effect of including kale or swedes in the diet of pasture-fed lactating dairy cows on chemical composition, fatty acid (FA) profile and sensory characteristics of Chanco-style cheese. Kale or swedes can be used in the diet of pasture-fed lactating dairy cows without negative effects on milk production, milk composition and cheese composition. However, with regard to cheese FA profiles, those elaborated from milks from kale and swedes increased total contents of saturated fatty acids.

**Abstract:**

Brassica crops such as kale and swede can be supplied to cow diets during winter, however little is known about the effects of feeding those forage brassicas to lactating cows on cheese nutritional characteristics of milk and cheese. This study evaluated the effect of including kale or swede in pasture-fed lactating dairy cow diets on chemical composition, fatty acid (FA) profile, and sensory characteristics of Chanco-style cheese. Twelve early-lactation cows were used in a replicated (*n* = 4) 3 × 3 square Latin square design. The control diet consisted of (DM basis) 10.0 kg of grass silage, 4.0 kg of fresh grass pasture, 1.5 kg soybean meal, 1.0 kg of canola meal, and 4.0 kg of cereal-based concentrate. The other treatments replaced 25% of the diet with swede or kale. Milk yield, milkfat, and milk protein were similar between treatments as were cheese moisture, fat, and protein. Swede and kale increased total saturated cheese FA while thrombogenic index was greater in swede, but color homogeneity and salty flavor were greater while ripe cheese aroma less than for kale. Kale or swede can be used in the diet of pasture-fed lactating dairy cows without negative effects on milk production, milk composition, or cheese composition. However, kale and swede increased total cheese saturated FA.

## 1. Introduction

Brassicas, such as kales (*Brassica oleracea* (L.) ssp. *acephala*) and swedes (*Brassica napus* (L.) ssp. *napobrassica*), are used to supply feeds to ruminants during winter [1], a season with low pasture growth in humid temperate regions [2]. They can offer high DM production and nutritional quality in a short period of time, which is related with high metabolisable energy (ME), water-soluble carbohydrates (WSC) and low content of neutral detergent fiber (NDF) [3,4]. Winter brassicas have been used successfully in sheep [4], dry cows [5] and lactating dairy cows [6].

The chemical composition of brassicas varies due to the leaf/bulb-stem ratio [3,7]. The crude protein (CP) content of leaves can range from 15–25% on a DM basis, whereas the bulb of swedes varies from 9 to16 % of the DM [4,8]. In terms of sugar content (raffinose, sucrose, glucose, fructose), swede bulbs are higher (32%) than whole plant kale (18%). The NDF ranges from 16.5 to 19.6% in swedes and, 27.1 to 32.8% for kale; whereas soluble fiber (SF) ranges from 24 to 38% [7]. The soluble fiber is mainly composed of pectins (7–9%), galactans, and β-glucans, among others [4,8,9].

The FA in the fat globules of bovine milk have 3 main origins: FA contained in the lipoproteins circulating in the blood (from the diet and ruminal digestion), the non-esterified fatty acids from body mobilization (bound to albumin), and FA synthesized *de novo* in the mammary gland [10]. The short-chain FA and medium-chain FA are mostly synthesized *de novo* in the mammary gland, whereas long-chain FA and very long-chain FA come from the lipids circulating in the blood and from the fat mobilized from body reserves [11]. Acetate is the main carbon source for most FA that are synthesized [12], thus changes in rumen fermentation patterns affect the FA profile of milk. The differences in nutrient concentrations among winter brassicas and grass pasture may result in different fermentation patterns in the rumen and supply of dietary FA.

Recent research has shown that fermentation of swedes results in lower acetate and greater proportions of butyrate and propionate [9], whereas kale offered at the same amount as grass pasture to dry cows resulted in lower proportions of propionate and greater butyrate [13]. Regarding the lipid fraction, brassicas have high contents of FA with >20 carbons, which is in agreement with previous research on different brassica species [14,15]. On the opposite, grass pastures contain high concentrations of *n* − 3 fatty acids, particularly a-linolenic acids, which lead to increase levels of polyunsaturated fatty acids (PUFA) in milk [16]. Supplementation with other brassica forages such as fodder rape and summer turnip has been found to modify milk FA profile [15], increasing the proportion of saturated fatty acids (SFA) compared with grass silage-based diets, however the inclusion of forage rape in the diet did not modify FA profile compared with alfalfa hay-based diets [17].

Brassica forages contain secondary compounds (e.g., S-methyl-cysteine sulphoxide [SMCO], glucosinolates and nitrates) that can alter organoleptic characteristics of milk [8], such as flavor and odor, as glucosinolates such as thiocyanate from brassicas have been found to pass into milk and producing flavor defects in bovine milk [18]. In summer brassicas, cheeses made with milk from cows fed turnip and rape were differentiated by increased odor, flavor, spiciness, bitterness, and acidity [15].

To the best of our knowledge, no studies have reported FA profile and sensory characteristics of cheeses from cows fed kale and swedes. We hypothesize that inclusion of winter brassicas in dairy cows diets increases the proportion of SFA and reduces unsaturated fatty acids (UFA). The aim of this study was to determine chemical composition, fatty acid profile and sensory characteristics of Chanco-style cheese from dairy cows fed winter brassica crops. Chanco-style cheeses were used in this study as they are one of the most important cheeses in the Chilean market and they are defined as semi hard and greasy cheeses [19].

## 2. Materials and Methods 

### 2.1. Animals and Treatments

Animal care and procedures were carried out according to the guidelines of the Animal Care Committee of the Universidad Austral de Chile (Approval Number: 237/2015). Twelve multiparous, early-lactation (60 ± 11 d) dairy cows (Holstein Friesian) were selected based on milk yield (30.3 ± 2.7 kg/d) and bodyweight (530 ± 27 kg). The experiment was carried out in 3 periods of 21 d, which consisted of a 14-d diet adaptation period and a 7-d sampling period. During the study, water was offered *ad libitum* and animals were housed in individual stalls. Table 1 shows the composition of dietary treatments offered. The control diet was formulated based on requirements of a 550 kg BW lactating cow producing 32 kg of milk according to [20] and consisted of (DM basis) 10.0 kg of grass silage, 4.0 kg of fresh grass pasture, 1.5 kg of solvent extracted soybean meal, 1.0 kg of mechanically extracted canola meal, and 4.0 kg of cereal based commercial concentrate. The other treatments replaced 25% of the diet (all ingredients were removed at the same proportion, except for the amount of soybean meal and canola meal that remained constant to keep the three diets isoenergetic and isonitrogenous.) with swedes cv. Aparima Gold or kale cv. Coleor. All feeds were weighed and offered individually for each cow. Kale and swede were sown in October and November 2016 in two adjacent 0.5 ha area at a density of 4 and 1.5 kg seed/ha, respectively. The crops were sown on two dates with a 20 days interval, in order to offer plant material with a similar stage of maturity throughout the experiment (150–180 days after emergence with a leaf:stem ratio: 65:35 and leaf:root ratio: 25:75 for kale and swede, respectively). Swedes were harvested manually and offered as whole plant, whereas kale was mechanically harvested and offered chopped with a particle size of 5 cm.

### 2.2. Dry Matter Intake, Milk Production and Composition

Orts were measured daily to determine daily dry matter intake (DMI) for each cow. Cows were milked at 07:00 and 16:00 h and milk yield was recorded at each milking with automatic milk meters (MM27BC, DeLaval, Tumba, Sweden). The average for the final week of each period is reported. Representative milk samples (200 mL) for am and pm milkings were collected three days in the last week each the experimental period for fat and protein analyses by infrared spectrophotometer (Foss 4300 Milko-scan, Foss Electric, Denmark). Milk samples for fatty acid profile analyses were pooled by day proportionally, according am and pm milk yield, in 15 mL Falcon tubes containing 30 mg of potassium dichromate and stored at −20 °C until further analyses.

### 2.3. Cheese Manufacture

On the last day of each sampling week, 4 L of milk were collected from each cow, weighting the production in the morning (60%) and afternoon (40%), 07:00 and 16:30 h, respectively. Milks of the same treatments were mixed, cooled in an ice bath and transported to a temperature of 4 °C to the Department of Animal Sciences of the Pontificia Universidad Católica de Chile, where the cheeses were made and subject for analysis of chemical composition, FA profile and sensory characteristics.

A direct acidified Chanco-style cheese manufacture was carried out on a 15-kg scale based on the protocol described by Seguel et al. [15]. The cheesemilk obtained from each treatment was pasteurized at 65 °C for 30 min and cooled to 4 °C. The pH of the milks was reduced to 5.8 using a 25% (*w*/*w*) citric acid solution and warmed to 31 °C. Cheesemilks were then supplemented with calcium chloride (77% purity, Dilaco, Santiago, Chile) at a rate of 3.9 g/15 kg and equilibrated for 3 min. A solution of commercial powder chymosin (20% *w*/*w* in deionized water, strength 1:10,000; Kyrein^®^, Santiago, Chile) was added at a rate of 15 g/15 kg cheesemilk and allowed to stand for 45 min. The curd was cut into 1 × 1 × 1 cm cubes using vertical and horizontal knife wires and healed for 3 min. The curd-whey mixture was stirred for 10 min and then cooked at a heating rate of 1 °C/3 min to 38 °C and then maintained at that temperature for 30 min under continuous stirring. The whey was completely drained from the vats during 20 min. The curd was then milled by hand and brine-salted with 300 mL of a sodium chloride solution (18% *w*/*v*) and left to equilibrate for 20 min. The salted curds were then transferred to 250-g molds and the cheeses were pressed for 14 h. Finally, cheeses were ripened for 21 d at a temperature of 10 °C and a relative humidity of 80%.

### 2.4. Chemical Analyses of Dietary Treatments and Cheeses

For feeds, the dry matter content was determined by weighing before and after drying with a forced-air oven at 60 °C for 48 h and thereafter at 105 °C for 12 h. For each diet sample, ash and ether extract (EE) were analysed according to [21] (ID 942.05 and ID 920.39 for ash and EE, respectively); Nitrogen content was determined by combustion (Leco Model FP-428 Nitrogen Determinator. Leco Corp. St. Joseph, MI, USA) and was used to calculate CP content (N × 6.25); neutral detergent fibre was determined as aNDF [22] using heat stable amylase (Ankom Technology Corp., Macedon, NY, USA); and acid detergent fibre according to [21] (ID 973.18). The sequential fibre analysis with correction for residual ash was conducted. The composition of experimental cheeses was determined at 21 d of ripening for moisture by the oven-drying method [21], fat by the Gerber method [23], total protein (N × 6.38) by Kjeldahl method [21] and salt by potentiometric method [24].

### 2.5. Milk and Cheese Fatty Acid Profile Analysis

Lipids from milk and Chanco-style cheese samples were extracted according to the method proposed by [25] and the methylation was performed according to the Christie protocol [26] with modifications by [27]. Then a gas chromatography system (Shimadzu Scientific Instruments AOC-20, Columbia, MD, USA) equipped with a 100 m column with the following chromatographic conditions was used: after the injection, the oven temperature was set at 110 °C for 4 min and after that it was raised to 160 °C at a rate of 5 °C/min for 10 min, then to 225 °C at a rate of 3 °C/min for 10 more minutes and finally increased to 240 °C at a rate of 3 °C/min. The temperature of the ionization flame was 260 °C, the injection volume 2 μL, the hydrogen flow 25 mL/min, the airflow 400 mL/min and the flow of nitrogen that makes up the gas was 40 mL/min. The fatty acid peaks in the gas chromatograph were identified using standardization methyl esters of fatty acids (FAME, Supelco 37 Component FAME mix, Bellefonte, PA, USA). Retention times were compared with those from similar studies focused on Chanco-style cheese FA profile [15,19].

### 2.6. Nutritional Evaluation of Cheese Fat

The nutritional quality of the FA contained in the milk and cheeses was evaluated as indicated by [28]. In this way, the atherogenic index and thrombogenic index (TI) were determined as: AI = [C12:0 + (4 C14:0) + C16:0/(PUFA *n* − 6 + PUFA *n* − 3) + MUFA] and TI = [(C14:0 + C16:0 + C18:0/0.5 MUFA) + (0.5 PUFA *n* − 6) + (3 PUFA *n* − 3) + (PUFA *n* − 3/PUFA) *n* − 6)]. For AI and TI, C12:0, C14:0, and C16:0 are considered as atherogenic FA while C14:0, C16:0, and C18:0 are considered as thrombogenic FA. Additionally, total *n* − 3 FA, total *n* − 6 FA, and the *n* − 6/*n* − 3 ratio were determined.

### 2.7. Sensory Analysis of Cheeses

Experimental cheeses at 21 d of ripening were subjected to a descriptive sensory analysis as described by Seguel et al. [15]. The sensory panel was comprised of 14-trained panelists who were not provided with any information about cheese treatments. Cheese samples cubes (1 × 1 × 1 cm) at 12 °C were evaluated based on attributes of appearance (color homogeneity and holes), aroma (milk aroma, overall aroma and ripe cheese aroma), texture (hardness, graininess, sound, moisture, adhesiveness) and taste, and flavor (salt, acid, bitter, spiciness before and after swallowing, overall flavor, ripe cheese flavor, and astringency). The judges evaluated the cheeses sequentially by rating the attributes on a continuous intensity scale from 0 (none) to 9 (pronounced).

### 2.8. Experimental Design and Statistical Analysis

According to the milk production, live weight, fat, protein and days in milk, four sub-groups of three animals (squares) were constituted and randomly assigned to one of the three treatments: Control, Swede and Kale. All animals went through each of the treatments in a replicated (*n* = 4) 3 × 3 square Latin square design and balanced for residual effects (three treatments and three periods of 21 days) as described by [29], where after period one, the animals were distributed to another treatment, taking into account that at the end of the experiment all possible treatment sequences were conducted to determine the presence of a carryover effect. As carryover effect was not detected, data from milk production and composition were analyzed using the mixed model procedure of SAS (Proc Mixed; SAS Institute, 2006, Kerry, NC, USA) to account for effects of square, period within square, cow within square, and treatment. The dietary treatment was considered a fixed effect; square, period within square, and cow within square were considered random effects.

To determine the FA profile contained in the cheeses, a completely randomized block design was used, where the block corresponded to the experimental period. Before carrying out the analysis of variance, the assumptions of normality were checked by the Kolmogorov-Smirnov test and homogeneity of variance with the Levene test. When statistical differences were observed (*p* < 0.05) the means were compared by the Tukey test.

A principal component analysis (PCA) using a correlation matrix was performed on the sensory attributes of experimental cheeses to identify groups of data related to the treatments evaluated. Multivariate analysis was carried out using Minitab^®^ 19 (Minitab Inc., State College, PA, USA).

## 3. Results and Discussion

### 3.1. Performance and Milk Composition

Milk yield (30 kg/d), milkfat (4.12 g/100 g) and milk protein (3.2 g/100 g) were similar between treatments (Table 2). Compared with control and swede, kale reduced dry matter intake (20.9 and 20.3 vs. 19.5 kg/d). Our DMI results are similar to those reported previously [15], where forage turnip and forage rape were fed at similar dietary inclusion rates to lactating cows. Reductions in DMI of about 16% have been reported with mid-lactating cows fed with turnips [30]. Our results on DMI could be explained by the high content of water present in kale that may have resulted in an increased rumen fill sensation (satiety) that led to a decrease in feed consumption [31,32]. From a farmer’s perspective, our findings on animal performance and milk components are relevant specially when there is a need for alternative winter forages to the common use of grass pasture and grass silages.

### 3.2. Fatty Acids in Milk

In milk, compared with control and kale, swede increased C18:3*n* − 3, C20:3*n* − 3, C22:6*n* − 3, and total *n* − 3. Both swede and kale decreased total monounsaturated fatty acids (FA) and tended (*p* = 0.052) to increase total saturated fatty acids (Table 3). Results on milk FA profiles mirrored the FA profiles shown previously in Table 1, where swede had greater *n* − 3 FA. In the rumen, dietary FA undergo a biohydrogenation process whereby bacteria convert dietary unsaturated FA to saturated FA [33]. Therefore, the rate of rumen biohydrogenation will depend on the type and amount of dietary lipid sources and if the dietary content of unsaturated FA exceeds the rumen bacteria capacity to saturate FA, with increasing amounts of FA with double bonds escaping the rumen and being secreted in milk will be increased [34].

It is worth mentioning that kale and swede did not increase contents of C18:1 trans-10 of which presence has been related to milk fat depression [35]. That partly explains why milkfat was not affected by dietary treatments. Also, in rumenic acid (C18:2 cis-9, trans-11) was not affected by dietary treatments. From a nutritional perspective this is desirable as in humans this FA has been reported to prevent cardiovascular diseases as it prevents atherosclerosis and inflammation [36].

With regard to the nutritional value of milk, compared with control and swede, kale increased thrombogenic index. The TI accounts for the FA that might have an effect on human health and, in particular, this index shows the tendency for blood clot formation in the blood vessels [28]. Specifically, this index is defined as the relationship between the pro-thrombogenic (such as saturated FA) and the anti-thrombogenic FA (such as *n* − 6 polyunsaturated FA) [37]. Thus, milk from kale may be of less benefit for human health.

Milk from swede were higher in C18:3n − 3 and C20:3n − 3 which were also higher in the FA profile from that treatment, therefore it could be inferred that the supply of these individual FA was of a magnitude that allowed their escape from rumen biohydrogenation. In humans, intake of alpha-linolenic acid (C18:3n − 3), has been shown to decrease the risk of cardiovascular disease as it reduces blood levels of triglycerides, cholesterol, high-density lipoprotein, low-density lipoprotein, and very-low-density lipoprotein [38]. Eicosatrienoic acid (C20:3n − 3) and docosapentaenoic acid (C22:6n − 3) were increased in milk from swedes, those FA belong to the so-called omega 3 FA and in humans they can have many benefits such as improving cognition and inflammation [39]. Total contents of *n* − 3 FA were higher in milk from swede. It has been suggested that *n* − 3 FA could help to promote a COVID-19 anti-inflammatory response when there is at least an intake of *n* − 3 FA of around 2.2 g/day for C18:3n − 3 and 500 mg/day for C20:5n − 3 + C22:6n − 3 [40].

Compared to control and swedes, an increased n − 6/n − 3 ratio from milk from kale was observed. However, according to the British Department of Health [41] the recommended value for this ratio is <4. Therefore, from this angle, milk from all treatments meet that recommendation.

In an overall perspective, milk from swedes may be healthier for human consumption.

### 3.3. Chemical Composition and Fatty Acids in Cheese

Chemical composition of cheeses was similar between treatments. Mean values for moisture, fat and protein were 45 g/100 g, 30 g/100 g and 21 g/100 g respectively. The lack of changes in cheese chemical composition was expected as the cheese manufacturing protocol aimed at analyzing the effect of lipids from forage brassicas and thus, milk used to elaborate cheeses was not standardized for fat content as has been done in previous studies [15,42]. Fat content for full-fat Chanco cheese should be of 25 g/100 g of cheese, as indicated by the Chilean norm [43]. The high content of fat observed in our experimental cheeses can be explain by the fat contents of the milks used for cheese manufacturing.

Cheese chemical composition is strongly influenced by the milk used for its manufacturing [44]. In a similar study [15] where summer brassicas were fed to lactating cows, FA profiles from milk and cheese were of similar values. Although, some individual cheese FA were affected by treatments, the magnitudes of change were similar to those observed for milk FA profiles.

Cheeses from swede and kale increased total saturated fatty acids and this was a reflection from the tendency (*p* = 0.062) of C16:0 to be increased as well as the tendency (*p* = 0.090) of total monounsaturated FA to decrease (Table 4). The TI was higher in swede. Detected contents of total saturated FA (70 g/100 g), total monounsaturated FA (23 g/100 g) and total polyunsaturated FA (4 g/100 g) were similar to other studies working with Chanco-style cheeses [15,19,38]. These values are expected in ruminant dairy products as dietary unsaturated FA will undergo a process known as biohydrogenation whereby rumen microorganisms (mostly bacteria) hydrogenate those FA and the end products are mainly saturated FA such as C18:0 [33,45]. This can clearly be seen in the preponderance of C14:0, C16:0, and C18:0 in cheeses from all treatments.

### 3.4. Sensory Characteristics

**Figure 1:**
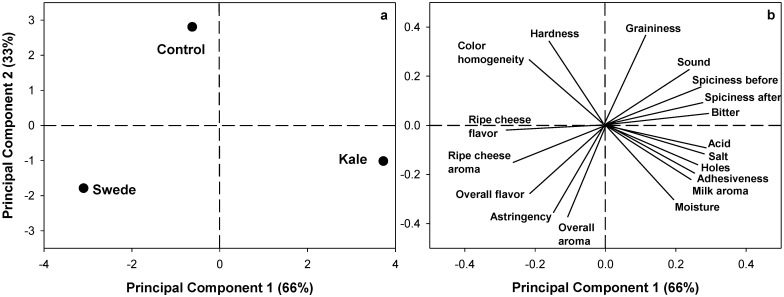
Score (**a**) and loading (**b**) plots obtained by principal component analyses (PCA) for all 18 sensory attributes of Chanco-style cheeses made with milks obtained from dairy cows fed with control diets or supplemented with swede or kale.

Sensory characteristics of Chanco-style cheese were similar between treatments. Color homogeneity and salty flavor obtained the higher notes while ripe cheese aroma obtained the lowest notes. Hardness texture tended (*p* = 0.069) to be lower in kale, while bitter taste tended (*p* = 0.073) to be lower in swede (Table 5). The score and loading plots obtained from the PCA of the descriptive sensory analysis is shown in Figure 1. Two components (Principal Component 1 and Principal Component 2) accounted for 99% of total variance (66 and 33%, respectively). The score plot (Figure 1a) showed that PC1 separated samples among treatments, whereas PC2 separated control sample from samples treated with swede and kale. Vectors loadings (Figure 1b) showed that Control and swede treatments were associated with increased hardness, color homogeneity, aromas, and flavors, whereas kale treatment was positively associated with attributes of salt, acid, milky notes, and spiciness.

The composition of experimental cheeses is in accordance with Chilean legislation regarding Chanco-style cheese (i.e., >44% moisture, >50% fat in dry matter and >61% moisture in nonfat substance) [23]. Similar composition of cheeses among treatments was achieved by similarities in the composition of milks used for cheesemaking (Table 2), as well as the use of a standardized manufacture protocol [46].

As shown in the PCA, cheeses from control and swede had higher notes for aromas and flavors, while cheese from Kale had greater milk aroma and acid and salty flavors and, more importantly, greater spiciness before and after swallowing. This shows that secondary compounds from kale were easily transferred to cheese, but the mechanisms are not well understood. Our previous study [15], also reported that feeding cows with either turnip or rape increased notes of bitterness and spiciness in cheese compared to that from a control diet. Another important point may be that the contents of plant sulphur compounds (such as methanethiol, carbon disulphide, and dimethyl-sulphide) from treatments are considered indispensable for cheese aroma [47]. Those plant compounds were not analyzed in this study. However, further efforts should be done to quantify them in the plant and in the final food matrix.

Overall, from a consumer perspective, this study has provided interesting results on the nutritional quality of milk and cheese from cows fed with winter forage brassicas. Today, consumers are aware about the biological repercussions of consuming some groups of FA such as the saturated FA that are the most predominant in dairy products fat [48]. In this regard, swedes and kale increased total saturated FA. Another feature from this study was the sensory characteristics from cheeses made from cows fed with swede and kale resulted to be similar (although bitter and spicy flavors deserve further attention).

On the other hand, from a farmer’s perspective, swedes and kale did not affect overall animal performance and milk composition, which can imply that such winter forages could be of great use in temperate regions, when there is a lack of conserved forage sources such as grass silage.

## 4. Conclusions

Kale or swede can be used in the diet of pasture-fed lactating dairy cows without negative effects on milk production, milk composition, or cheese composition. However, with regard to cheese FA profiles, those elaborated from milks from kale and swede increased total contents of saturated fatty acids which also led to an increase in the thrombogenic index. Thus, for farmers and the dairy industry, caution must be paid to the use of both winter brassicas if bioactive cheese fatty acids are sought.

## Figures and Tables

**Table 1:** Nutrient concentration and fatty acids profile (g/100 g of total fatty acids) of the diets offered to early lactation dairy cattle producing milk to be processed into Chanco-style cheese.

	Control	Swedes	Kale
DM (g kg −1)	485	388	395
Ash (g kg −1 DM)	85	99	98
CP (g kg −1 DM)	190	193	193
aNDF (g kg −1 DM)	367	312	335
ADF (g kg −1 DM)	180	163	179
Lipids (g kg −1 DM)	45	37	40
NFC (g kg −1 DM)	312	359	335
ME (Mcal kg −1 DM)	2.91	2.93	2.90
Fatty acids (g/100 g of total fatty acids)			
C16:0	5.12	14.88	6.56
C17:0	ND	ND	ND
C18:0	9.79	2.69	2.73
C18:1n9c	41.80	26.01	23.79
C18:2n6t	ND	4.90	14.94
C18:2n6c	29.08	7.83	18.83
C18:3n6	ND	ND	9.69
C18:3n3	11.40	20.00	11.51
C20:5n3	2.81	23.69	11.95
Σ SFA	14.91	17.57	9.29
Σ MUFA	41.80	26.01	23.79
Σ PUFA	40.48	56.42	66.92

SFA; saturated fatty acids; MUFA: mono unsaturated fatty acids; PUFA: polyunsaturated fatty acids; ND: not detected.

**Table 2:** Dry matter intake, milk production and milk composition by early lactating dairy cows fed a control diet or with kales or swedes.

	Treatments			SEM	*p*-Value
	Control	Swede	Kale		
Intake					
Brassica intake, kg/DM/day	-	4.3	2.8	0.22	0.002
Dry matter intake, kg/DM/day	20.9 ^a^	20.3 ^a^	19.5 ^b^	0.31	0.003
Production and composition					
Milk yield, kg/day	30.3	30.7	30.1	0.95	0.642
Fat, g/100 g	4.19	4.15	4.04	0.12	0.411
Crude protein, g/100 g	3.20	3.21	3.19	0.05	0.505
4% Fat-corrected milk, kg/day	31.4	31.2	30.2	1.20	0.291

^a,b^ Means in the same row with different superscripts differ significantly for treatment effect with the *p*-value shown; SEM = Standard error of the mean.

**Table 3:** Fatty acid profile (g/100 g of total fatty acids) of milk from early lactation dairy cows fed control or supplemented with swedes or kale.

Fatty Acid	Control	Swede	Kale	SEM	*p*-Value
C4:0	3.21	3.16	3.45	0.116	0.189
C6:0	2.38	2.26	2.32	0.587	0.352
C8:0	1.37	1.32	1.31	0.058	0.719
C10:0	2.75	2.58	2.89	0.145	0.322
C11:0	0.37	0.33	0.29	0.042	0.507
C12:0	3.22	3.49	3.22	0.152	0.349
C13:0	0.28 ^a^	0.22^a^	0.11 ^b^	0.039	0.018
C14:0	11.9	12.8	12.0	0.282	0.056
C14:1	0.73	0.64	0.70	0.069	0.659
C15:0	0.75	0.77	0.68	0.086	0.735
C15:1	0.33	0.38	0.16	0.066	0.076
C16:0	31.6	31.5	32.7	0.614	0.317
C16:1	0.77	0.89	0.70	0.095	0.363
C17:0	0.48	0.45	0.39	0.059	0.533
C17:1	0.32	0.38	0.36	0.029	0.312
C18:0	13.4	14.1	14.4	0.546	0.413
C18:1 trans-10	0.14	0.05	0.07	0.046	0.410
C18:1 trans-11	0.40	0.34	0.31	0.070	0.686
C18:1 cis-9	21.2	19.5	19.7	0.639	0.162
C18:2n − 6 trans	0.56 ^ab^	0.62 ^a^	0.45 ^b^	0.037	0.015
C18:2n − 6 cis	0.46	0.46	0.43	0.039	0.813
C20:0	0.02	0.005	0.02	0.008	0.286
C20:1n − 9	0.01	0.009	nd	0.007	0.236
C18:3n − 6	0.48	0.43	0.26	0.083	0.190
C18:3n − 3	0.32 ^b^	0.70 ^a^	0.47 ^b^	0.081	0.014
C18:2 cis-9, trans-11	1.55	1.26	1.61	0.107	0.064
C20:3n − 3	0.12 ^b^	0.31 ^a^	0.18 ^b^	0.043	0.018
C20:3n − 6	0.19	0.16	0.17	0.054	0.919
C20:4n − 6	0.15	0.20	0.12	0.035	0.323
C20:5n − 3	0.06	0.09	0.06	0.032	0.673
C22:6n − 3	0.06 ^b^	0.21 ^a^	0.08 ^b^	0.033	0.009
Σ Saturated fatty acids	71.9	73.2	74.0	0.560	0.052
Σ Monounsaturated fatty acids	24.0 ^a^	22.3 ^b^	22.0 ^b^	0.542	0.050
Σ Polyunsaturated fatty acids	3.97	4.47	3.86	0.220	0.133
Σ *n* − 3	1.27 ^b^	1.93 ^a^	1.24 ^b^	0.113	<0.001
Σ n − 6	1.70	1.89	1.48	0.165	0.241
n − 6/n − 3	1.35 ^a^	0.97 ^b^	1.15 ^ab^	0.059	0.001
Atherogenic index	1.69	1.64	1.80	0.088	0.415
Thrombogenic index	2.39 ^b^	2.27 ^b^	2.52 ^a^	0.068	0.047

^a,b^ Means in the same row with different superscripts differ significantly for treatment effect with the *p*-value shown; SEM = Standard error of the mean; nd = not detected; *n* − 3 = omega 3 fatty acids; *n* − 6 = omega 6 fatty acids.

**Table 4:** Composition (g/100 g) and fatty acid profile (g/100 g of total fatty acids) of Chanco-style cheeses made with milks from early lactation dairy cows fed control or supplemented with kale or swedes.

	Control	Swede	Kale	SEM	*p*-Value
Cheese composition					
Moisture	45.2	45.2	44.6	0.493	0.142
Fat	29.4	29.7	30.1	0.245	0.252
Protein	21.7	20.9	20.8	0.388	0.274
Salt	1.36	1.30	1.30	0.031	0.401
Moisture in nonfat substance	64.0	64.4	63.8	0.264	0.375
Fat in dry matter	53.7	54.3	54.4	0.401	0.459
Salt in moisture phase	3.01	2.88	2.81	0.073	0.496
Fatty acid					
C4:0	3.56	3.67	4.10	0.298	0.468
C6:0	2.40	2.47	2.73	0.192	0.509
C8:0	1.37	1.40	1.50	0.120	0.745
C10:0	3.59	3.26	3.27	0.194	0.461
C11:0	0.31	0.32	0.34	0.027	0.719
C12:0	4.01	3.68	3.52	0.248	0.438
C13:0	0.19	0.09	0.09	0.048	0.322
C14:0	13.3	12.4	11.3	0.592	0.158
C14:1	0.95	1.00	0.87	0.105	0.530
C15:0	0.91	0.83	0.63	0.154	0.423
C15:1	0.12	0.11	0.12	0.010	0.749
C16:0	28.0	35.2	33.3	1.847	0.062
C16:1	1.38	1.12	1.34	0.201	0.641
C17:0	0.38	0.34	0.33	0.035	0.531
C17:1	0.45	0.35	0.32	0.046	0.225
C18:0	13.0	11.4	11.2	0.920	0.201
C18:1 trans-10	0.03	0.06	0.03	0.026	0.244
C18:1 trans-11	0.64	0.50	0.45	0.225	0.749
C18:1 cis-9	21.87	17.66	20.0	1.272	0.137
C18:2n − 6 trans	0.46	0.88	1.08	0.167	0.083
C18:2n − 6 cis	0.52	0.72	0.78	0.389	0.735
C18:3n − 6	0.03	0.47	0.38	0.172	0.269
C18:3n − 3	0.59	0.49	0.60	0.103	0.734
C18:2 cis-9, trans-11	1.14	1.01	0.67	0.120	0.106
C20:0	0.10	0.05	0.04	0.050	0.410
C20:1n − 9	0.009	0.01	0.06	0.023	0.187
C20:2	0.04	0.005	nd	0.004	0.444
C22:0	0.01	0.02	0.02	0.018	0.444
C20:3n − 3	0.12	0.03	0.18	0.077	0.432
C20:3n − 6	0.13	0.05	0.15	0.073	0.638
C20:4n − 6	0.11	0.07	0.17	0.081	0.643
C20:5n − 3	0.02	0.03	0.03	0.026	0.953
C22:6n − 3	0.03	0.07	0.15	0.043	0.223
Σ Saturated fatty acids	67.4 ^b^	72.3 ^a^	70.8 ^a^	1.221	0.046
Σ Monounsaturated fatty acids	25.0	20.4	22.8	1.048	0.090
Σ Polyunsaturated fatty acids	3.18	3.87	4.25	0.620	0.414
Σ n − 6	1.26	2.22	2.59	0.473	0.132
Σ n − 3	0.77	0.63	0.98	0.176	0.449
n − 6/n − 3	1.69	3.81	2.87	0.837	0.306
Atherogenic index	1.92	2.08	1.68	0.219	0.114
Thrombogenic index	2.40 ^b^	3.06 ^a^	2.60 ^b^	0.169	0.040

^a,b^ Means in the same row with different superscripts differ significantly for treatment effect with the *p*-value shown; SEM = Standard error of the mean; nd = not detected; *n* − 3 = omega 3 fatty acids; *n* − 6 = omega 6 fatty acids.

**Table 5:** Sensory characteristics of Chanco-style cheeses made with milk from dairy cows fed control or supplemented with kale or swedes.

	Treatments				
Attribute	Control	Swede	Kale	SEM	*p*-Value
Appearance					
Color homogeneity	6.59	6.44	6.20	0.427	0.794
Holes	4.16	4.24	5.59	0.555	0.238
Aroma					
Milk aroma	3.61	3.63	3.71	0.269	0.960
Overall aroma	3.56	4.43	3.98	0.293	0.227
Ripe cheese aroma	2.90	3.00	2.85	0.119	0.692
Texture					
Hardness	4.85	4.24	3.82	0.220	0.069
Graininess	4.94	4.49	4.74	0.208	0.392
Sound	4.68	3.85	4.75	0.206	0.065
Moisture	3.57	3.99	4.50	0.212	0.084
Adhesiveness	3.39	3.44	3.79	0.298	0.628
Taste and flavor					
Salt	5.93	5.90	6.18	0.414	0.868
Acid	4.23	4.14	4.88	0.200	0.105
Bitter	3.18	2.58	3.78	0.275	0.073
Spiciness before	3.38	2.82	3.65	0.269	0.196
Spiciness after	3.38	3.04	3.64	0.291	0.431
Overall flavor	4.97	5.30	4.99	0.133	0.267
Ripe cheese flavor	3.82	3.99	3.57	0.184	0.355
Astringency	3.35	3.55	3.43	0.209	0.811

SEM = Standard error of the mean.

## Data Availability

The data presented in this study are available on request from the corresponding author.
